# Avian UV vision enhances leaf surface contrasts in forest environments

**DOI:** 10.1038/s41467-018-08142-5

**Published:** 2019-01-22

**Authors:** Cynthia Tedore, Dan-Eric Nilsson

**Affiliations:** 1 Lund Vision Group, Lund University, Sölvegatan 35, Lund, 223 62 Sweden; 2 Present Address: Zoological Institute, University of Hamburg, Martin-Luther-King Platz 3, Hamburg, 20146 Germany

## Abstract

UV vision is prevalent, but we know little about its utility in common general tasks, as in resolving habitat structure. Here we visualize vegetated habitats using a multispectral camera with channels mimicking bird photoreceptor sensitivities across the UV-visible spectrum. We find that the contrast between upper and lower leaf surfaces is higher in a UV channel than in any visible channel, and that this makes leaf position and orientation stand out clearly. This was unexpected since both leaf surfaces reflect similarly small proportions (1–2%) of incident UV light. The strong UV-contrast can be explained by downwelling light being brighter than upwelling, and leaves transmitting < 0.06% of incident UV light. We also find that mirror-like specular reflections of the sky and overlying canopy, from the waxy leaf cuticle, often dwarf diffuse reflections. Specular reflections shift leaf color, such that maximum leaf-contrast is seen at short UV wavelengths under open canopies, and at long UV wavelengths under closed canopies.

## Abstract (web summary)

The utility of UV vision for visualizing habitat structure is poorly known. Here, the authors use optical models and multispectral imaging to show that UV vision reveals sharp visual contrasts between leaf surfaces, potentially an advantage in navigating forest environments.

## Introduction

Most animals with color vision have UV vision^1^. However, the full range of tasks that UV vision may be adapted for remains poorly explored due to the difficulty in making predictions about a waveband that we cannot see. Most of what we know about the utility of UV vision comes from targeted, species-specific investigations of animal and flower color signals, and from inferences that figure-ground contrast must be strong for objects seen against the UV-bright sky or shallow-water space light^1^. We also know that the sky is a strong emitter of polarized light, and that animals using skylight polarization patterns for navigation generally have polarization-sensitive photoreceptors peaking in the UV or blue^2^. By contrast, little is known about the utility of UV vision for visualizing contrasts between common terrestrial objects encountered by most land-based animals in their everyday activities. One reason we lack such knowledge is that it is difficult to predict what object comparisons might generate high UV contrasts when all of the objects in question (e.g., leaves, trunks, leaf litter, rocks, etc.) are known to reflect little UV light^3,4^.

To circumvent these roadblocks to hypothesis generation, we made use of an underutilized technology in the field of visual ecology—multispectral imaging. We developed camera channels that mimicked real animal spectral sensitivities, which enabled us to visualize the raw data collected by photoreceptors. This technique compensated for our own UV-blindness, and allowed us to quantitatively assess the relative benefit of differently tuned photoreceptor channels across the UV-visible spectrum. It also allowed us to observe habitats as they occur, in situ, without disturbing natural lighting geometry.

We adopted avian tetrachromatic vision as a model system. Bird vision is an excellent model system for several reasons. First, terrestrially foraging birds spend much of their time flying and/or hopping through vegetation, and many search specific leaf surfaces for prey^5^. Birds sample the entire UV-visible spectrum, from UV to red^6^, which makes them a good system for comparing a UV channel with the full range of visible channels. Birds also vary in their UV-cone spectral sensitivities^7–9^, which allowed us to test whether habitat type may drive the fine-tuning of UV spectral sensitivities in birds and other animals.

We find that leaf optics and habitat lighting geometry interact to make UV vision particularly adaptive for visualizing leaf surface contrasts. A UV channel should thus facilitate navigation through complex leafy habitats, as well as the localization of particular leaf surfaces for various tasks, including prey searching, oviposition, and refuge seeking. Using optical modeling, we show that specular reflections in different habitats play an underappreciated role in shaping the apparent color of leaves and, in turn, leaf surface contrasts. For visualizing leaf surface contrasts, we show that closed canopies favor longer-wavelength UV photoreceptors, whereas open canopies favor shorter-wavelength UV photoreceptors. This result may have implications for the spectral tuning of animal photoreceptors.

## Results and discussion

**Fig. 1:**
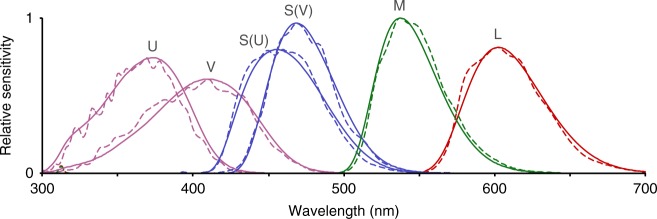
Spectral sensitivities of avian cones and multispectral camera channels. Solid lines show spectral sensitivities of avian cones and dashed lines show multispectral camera channels. Most terrestrially foraging birds are tetrachromats, having L, M, and either S(U) and U or S(V) and V cones^6^. L, M, S, V, and U stand for Long, Medium, Short, Violet, and Ultraviolet wavelengths, respectively

We used an avian-vision multispectral camera to visualize within-channel contrasts, comparing two common UV-cone variants (U and V, often referred to in the literature as UVS and VS^9^) to two common S (blue)-cone variants and M (green)- and L (red)-cones (Fig. 1). We also compared the two UV-cones with each other. Each UV-cone variant is typically accompanied by a specific blue-cone variant^9^, so we also compared the two blue-cone variants to each other. Specifically, we (1) looked for sources of high UV-contrast in vegetated habitats that are missing or weaker in other color channels, (2) tested how UV-contrast contributes to color contrast in tetra-, tri-, and di-chromatic visual systems, and (3) tested whether habitat type influences optimal spectral tuning of UV- and blue photoreceptor spectral sensitivities.

Images were classified into three habitat types—(1) rangelands and forest fragments dominated by deciduous vegetation in Skåne, Sweden, (2) old-growth wet schlerophyll forests having an understory of rainforest shrubs overshadowed by a tall, open eucalypt canopy in Queensland, Australia, and (3) old-growth tropical and subtropical rainforests in Queensland, Australia. The three habitats formed a progression from open, sparsely vegetated habitats, to closed, densely vegetated habitats.

**Fig. 2:**
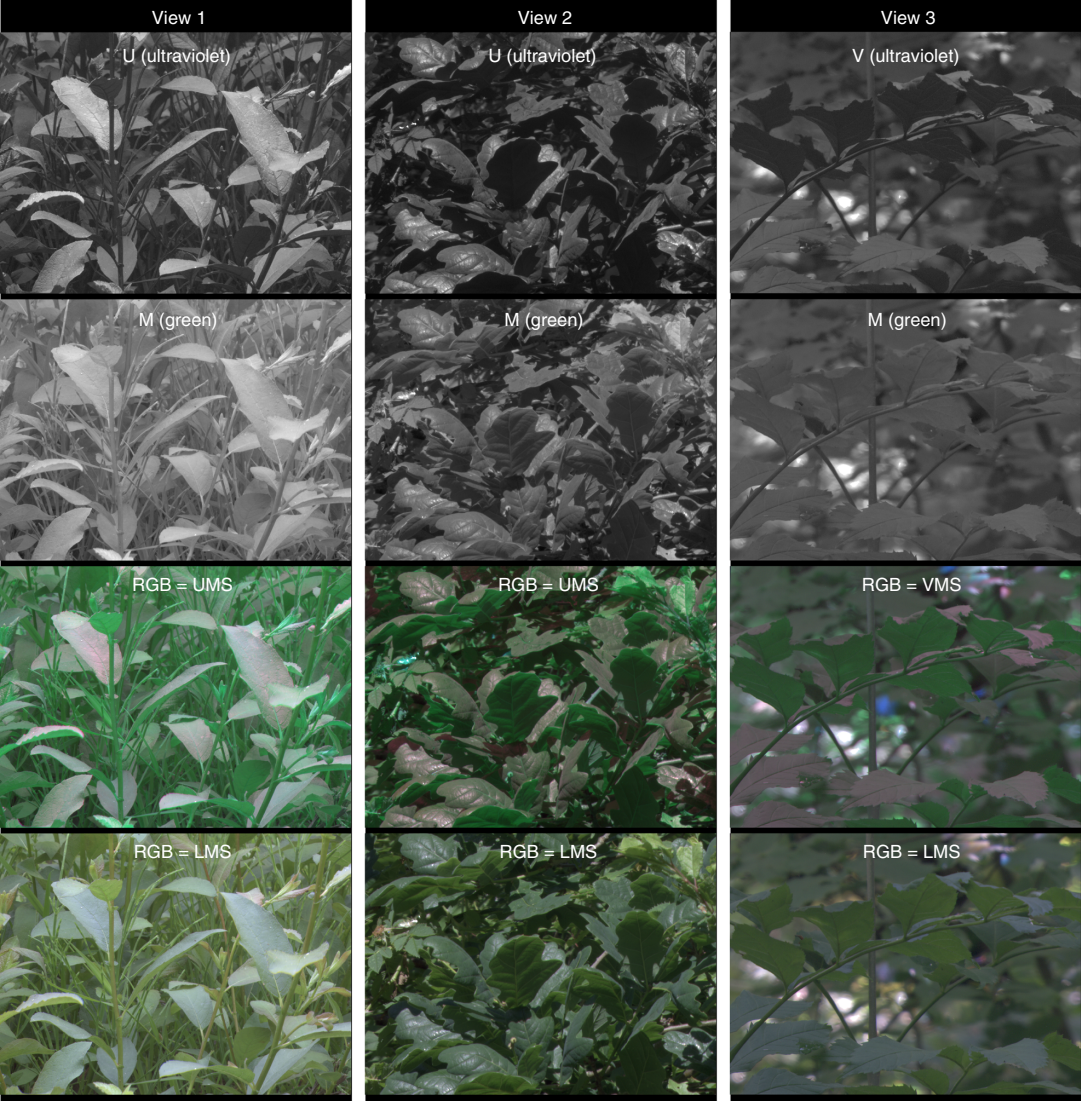
Example images showing the difference in leaf-contrast seen by the UV- and green-cones in deciduous habitats in southern Sweden. For each of the three views, the top two images show the same scene as viewed by the UV and green photoreceptor channels. The UV-cone variant that saw the higher contrast is the one that is displayed. The bottom two images are false-color RGB images with three of the four avian cone channels plugged into the R (red), G (green), and B (blue) channels of a digital display. For example, RGB = UMS denotes that the U-cone is displayed by the red channel of an RGB display, the M-cone by the green channel, and the S-cone by the blue channel. Note that the upper row of RGB images contains a UV channel and the lower row does not. For display purposes, pixel values from original captured images have been (1) normalized by the mean pixel value in each image, (2) adjusted to fill the dynamic range of an sRGB digital display, and (3) adjusted to undo the sRGB gamma scaling of most digital display devices. This means that, viewed on an sRGB-calibrated display, within each channel, pixel intensity scales linearly with cone output. If a pixel was overexposed or underexposed in any channel, the corresponding pixel in all channels was set to white (for overexposed) or black (for underexposed), and excluded from all calculations. Note that false-color images cannot replicate what animals actually see, but provide the best approximation available

**Fig. 3:**
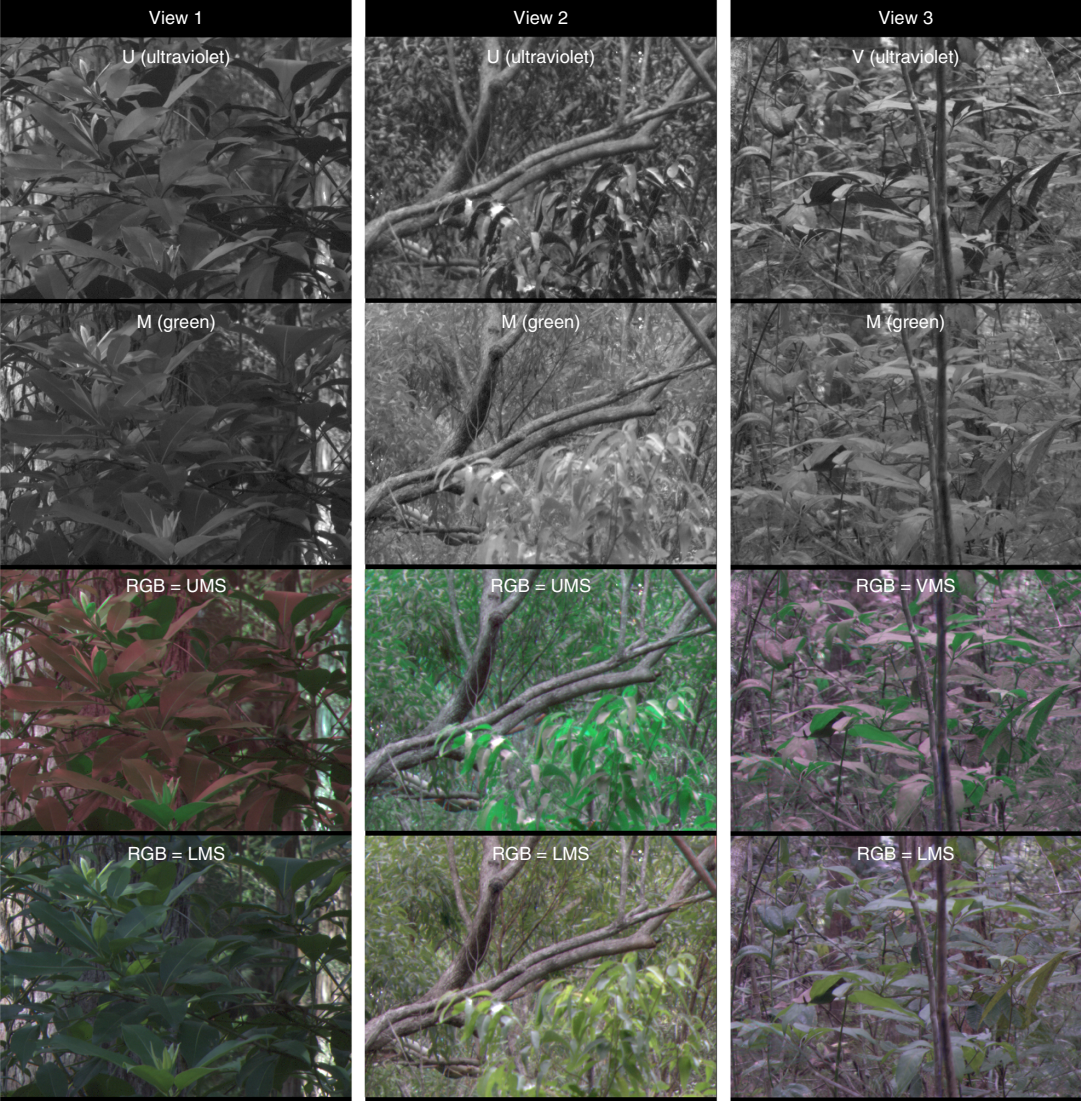
Example images showing the difference in leaf-contrast seen by the UV- and green-cones in wet schlerophyll habitats in Queensland, Australia. See Fig. 2 legend for further details

**Fig. 4:**
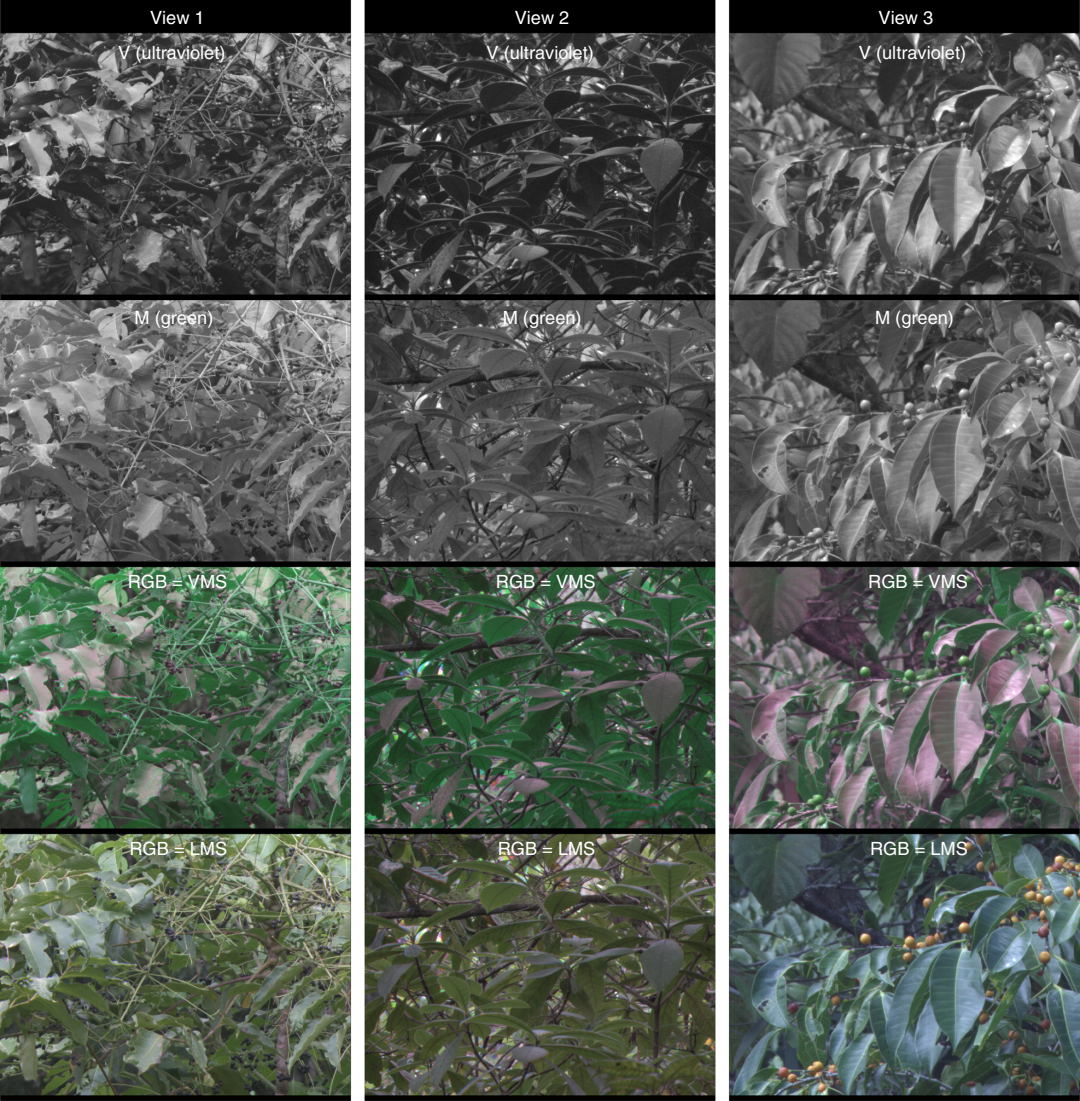
Example images showing the difference in leaf-contrast seen by the UV- and green-cones in rainforest habitats in Queensland, Australia. See Fig. 2 legend for further details

**Fig. 5:**
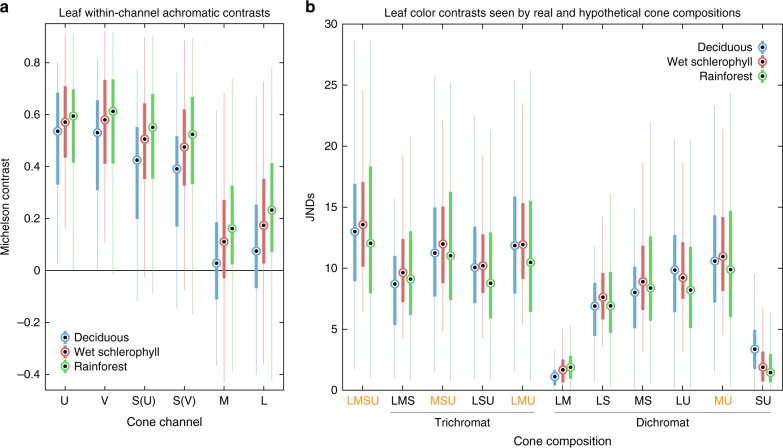
The advantage of UV vision for visualizing leaf achromatic and color contrasts. Boxplots show the median, interquartile range (IQR), and lowest and highest data, within 1.5 IQR, below and above the IQR (outliers not shown). **a** Michelson^10^ leaf achromatic contrasts seen by the different cone channels in different habitats. In each habitat, UV-cones saw higher leaf-contrast than all other cone channels at *P* < 0.0001. **b** Color contrasts in units of just noticeable differences (JNDs)^11^ seen by a LMSU tetrachromat and all possible trichromatic and dichromatic combinations of avian photoreceptors, again separated by habitat. To make our results as general as possible across the animal kingdom, all photoreceptor compositions were assumed to have the same number of each photoreceptor class in an integrative unit. In each habitat, transforming a trichromat in the visible range (LMS) into a tetrachromat with a UV-cone (LMSU) enhanced color contrast at *P* < 0.0001. All cone compositions containing both a U- and M-cone (highlighted in orange) saw higher leaf color contrast than any other composition having the same number of cones at *P* < 0.0001. All *P* values were derived from paired two-sided sign tests, i.e., “signtest” in MATLAB. Details of statistical tests can be found in Table 1

We found an obvious source of UV-contrast that was missing or weaker in the visible cone channels—the contrast between upper and lower leaf surfaces (henceforth leaf-contrast; calculated as Michelson contrast^10^) (Figs. 2–4, 5a, Table 1). Lower leaf surfaces were dark in the UV, their silhouettes enhancing the visibility of leaves and leaf edges and the geometry of the vegetation structure. This enhanced structural detail can be expected to increase efficiency in navigating vegetated habitats and in localizing particular leaf surfaces for hiding, oviposition, and prey searching.

**Table 1:** Details of sign tests used to analyze data in Fig. 5

Null hypotheses tested	Deciduous (*N* = 95) sign statistic, *P*-value	Wet schlerophyll (*N* = 109) sign statistic, *P*-value	Rainforest (*N* = 161) sign statistic, *P*-value
U-contrast−S(U)-contrast = 0	83, <0.0001	94, <0.0001	116, <0.0001
V-contrast−S(V)-contrast = 0	92, <0.0001	109, <0.0001	153, <0.0001
U-contrast−M-contrast = 0	94, <0.0001	109, <0.0001	160, <0.0001
V-contrast−M-contrast = 0	94, <0.0001	109, <0.0001	160, <0.0001
U-contrast−L-contrast = 0	94, <0.0001	109, <0.0001	156, <0.0001
V-contrast−L-contrast = 0	95, <0.0001	109, <0.0001	158, <0.0001
LMSU−LMS = 0	95, <0.0001	109, <0.0001	161, <0.0001
MSU−LMS = 0	90, <0.0001	109, <0.0001	148, <0.0001
MSU−LSU = 0	77, <0.0001	93, <0.0001	144, <0.0001
LMU−LMS = 0	84, <0.0001	94, <0.0001	117, <0.0001
LMU−LSU = 0	89, <0.0001	109, <0.0001	145, <0.0001
MU−LM = 0	95, <0.0001	109, <0.0001	157, <0.0001
MU−LS = 0	90, <0.0001	109, <0.0001	146, <0.0001
MU−MS = 0	84, <0.0001	94, <0.0001	117, <0.0001
MU−LU = 0	77, <0.0001	93, <0.0001	142, <0.0001
MU−SU = 0	92, <0.0001	109, <0.0001	155, <0.0001

The sign statistic indicates the number of times the difference in leaf-contrast seen by different channels or combinations of channels (shown in the null hypotheses tested column) was greater than zero

The green channel was surprisingly unhelpful for visualizing leaf-contrast (Figs. 2–4, 5a, Table 1). This challenges the natural intuition that because leaves are green, a green channel should be best for resolving vegetation structure. The striking difference between the green and UV channels can be explained by the fact that leaves transmit and reflect similar amounts of green light (and red and to a lesser extent, blue), but reflect much more UV light than they transmit^3^. In nature, downwelling illumination is much brighter than upwelling illumination, which translates into more light being available to reflect off of upper leaf surfaces than lower ones. These factors combine to make lower leaf surfaces radiate similar amounts of green, but very different amounts of UV light.

More specifically, in our measurements of upper leaf surface diffuse reflectance and transmittance in the 300–500 nm range (see Methods), we found that leaves diffusely reflect > 25 times as much UV light (300–400 nm) as they transmit, but reflect as little as 1.5 times as much blue light (400–500 nm) as they transmit. Previous work has shown that leaves reflect and transmit similar amounts of green (500–600 nm) and red (600–700 nm) light, with reflectance varying from ~0.7 to 1.3 times transmittance^3^.

In addition to enhancing within-channel contrast, we found that, in each habitat, transforming a trichromat in the visible range (LMS) by adding a UV channel to make a tetrachromat (LMSU) also enhanced leaf color contrast (Fig. 5b, Table 1). This was not surprising, as adding a color channel generates a new dimension along which color can vary. If we assume no cost in terms of increased noise in the pre-existing color channels, an additional color channel will always enhance color contrast if the objects being compared stimulate the new channel even by only slightly different amounts^11^. What was more interesting was that all theoretical trichromats and dichromats having both UV- and M-photoreceptors saw higher leaf color contrast than any other photoreceptor compositions having the same number of photoreceptor channels (Fig. 5b, Table 1). Most terrestrial vertebrate and invertebrate trichromats and dichromats have photoreceptors peaking in the same parts of the spectrum as avian UV- and M-cones^12–14^. Even among mammals, UV sensitivity has recently been found to be relatively common, with diverse species known, or predicted from opsin sequences, to have UV-peaking cones^15^, and with many known to have lenses transparent to UV wavelengths, enabling even blue (~400–450 nm)-peaking cones to be stimulated by UV wavelengths^16^. Our work demonstrates one possible reason why UV-green contrast is such a widely-sampled source of contrast across the animal kingdom.

**Fig. 6:**
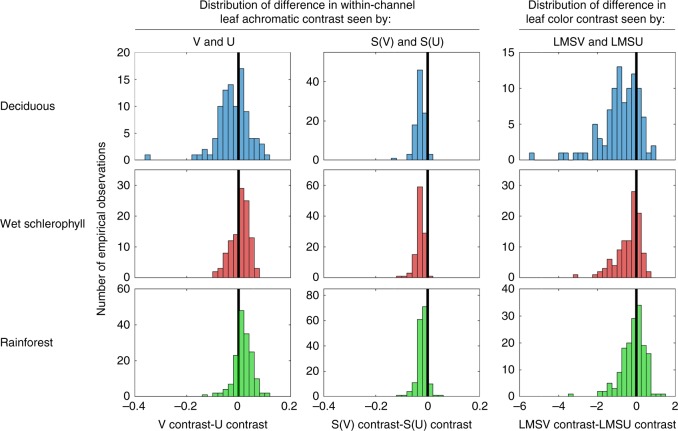
Multispectral camera data show that optimal spectral tuning of the UV-photoreceptor for visualizing leaf contrast depends on habitat type. Each of the left panels shows the distribution of the difference in within-channel leaf-contrast seen by the two UV-cone variants in a different habitat. The middle panels show the same distribution types for the two S-cone variants. The right panels show the same distribution types for color contrasts seen by LMSV and LMSU tetrachromats. The heavy vertical black line at *x* = 0 indicates the point at which the two variants see equal contrast. The V-cone was advantageous for visualizing leaf achromatic contrast in rainforest (*P* < 0.0001) and wet schlerophyll (*P* = 0.004) habitats, and the S(U)-cone was advantageous for visualizing leaf achromatic contrast in all habitats at *P* < 0.0001. An LMSU tetrachromatic system was advantageous for visualizing leaf color contrast in deciduous (*P* < 0.0001) and wet schlerophyll (*P* < 0.0001) habitats (paired two-sided sign tests, i.e., “signtest” in MATLAB; details of statistical tests in Table 2). In this figure, we used realistic relative photoreceptor abundances from terrestrially foraging birds such that the ratio of L:M:S:U cones in an integrative unit equaled 3:2.5:1.7:1^22–28^

We next tested for an effect of habitat type on optimal spectral tuning of the UV- and S-cones. We found that the V-cone was advantageous for visualizing leaf achromatic contrast in rainforest and wet schlerophyll habitats, whereas the S(U)-cone was advantageous for visualizing leaf achromatic contrast in all habitats (Fig. 6, Table 1). In an LMSV tetrachromat, if we swapped the V-cone for a U-cone, or the S(V)-cone for an S(U)-cone, one at a time, the effect on color contrast was very similar to the effect on within-channel achromatic contrast. However, as the U- and S-cone variants that co-occur do not have the same directional effects on within-channel contrast, their concerted effect on color contrast reflected a middle ground between their individual effects. The end result was that an LMSU tetrachromatic system (with S(U)- and U-cones) was advantageous for visualizing leaf color contrast in deciduous and wet schlerophyll habitats (Fig. 6, Table 1).

The fact that the S(U)-cone nearly always saw higher leaf-contrast than the S(V)-cone, regardless of habitat, suggests that any spectral tuning of the S-cone is likely directed by another evolutionary force unrelated to the perception of leaf-contrast. On the other hand, the fact that different UV-cone variants saw maximum leaf-contrast in different habitats may explain, in part, why birds have repeatedly flipped back and forth between two differently tuned UV-cone variants over their evolutionary history^7–9^, and may help explain variation in UV sensitivity in other groups of animals as well.

To mathematically validate the effect of habitat, and unravel the physics behind it, we developed an optical model to predict and compare leaf-contrast as seen by the V- and U-cones in different habitats. We measured the spectral properties of both deciduous and rainforest leaves, and tested both leaf types in the model. We designed the model to be robust to environmental variation by programming it to repeat its calculations 10,000 times, each time randomizing up to 12 different habitat and environmental parameters (for the parameter list, see Methods: Optical Model Calculations). The distribution of the difference in contrast seen by the V- and U-cones across the 10,000 repetitions of the model could then be visualized in a histogram. Comparing histograms generated from different combinations of parameters allowed us to validate the effect of habitat observed in our multispectral data. Once validated, we could then plot spectra generated during intermediate steps of the model to learn about the optical mechanisms behind the effect. In each of the model runs described below, all 12 parameters can be assumed to have been randomized unless otherwise specified.

**Fig. 7:**
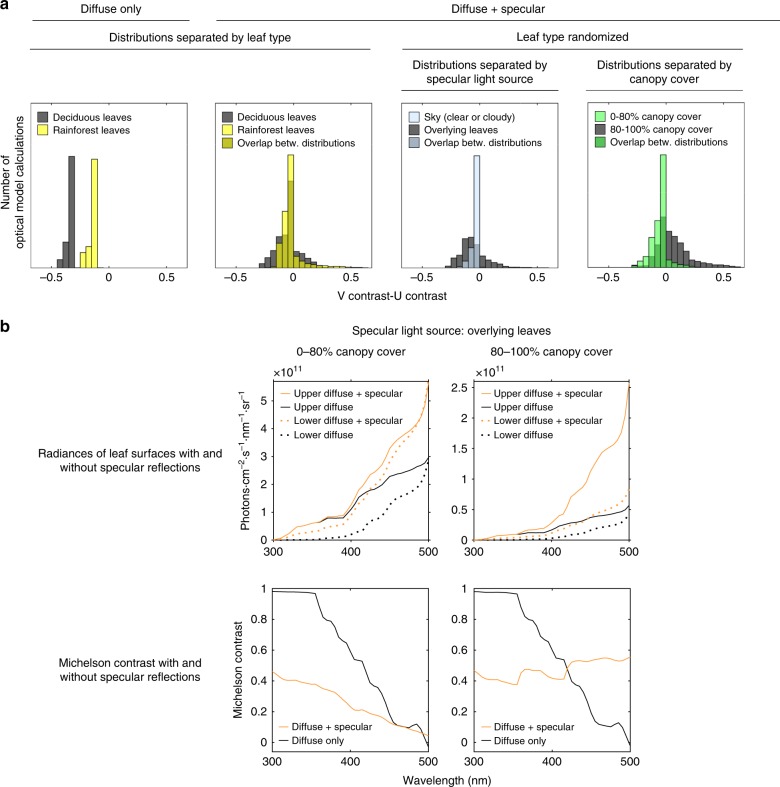
Optical model validates the effect of habitat observed in multispectral data and reveals the mechanism behind it. **a** First panel: when only diffuse reflectance and transmittance were modeled, the U-cone always saw higher leaf-contrast, regardless of whether deciduous or rainforest leaf spectra were used. Second panel: when specular reflections were added to the model, the V-cone sometimes saw higher leaf-contrast, but there was no clear separation between deciduous and rainforest leaf types. Third panel: by plotting leaves illuminated by different specular light sources, we found that the V-cone saw higher leaf-contrast only when overlying leaves were the specular light source. Fourth panel: comparing habitats with different amounts of overlying canopy demonstrated a clear separation between open and closed habitats, with the V-cone seeing higher leaf-contrast in closed habitats ( >80% canopy cover), and the U-cone seeing higher leaf-contrast in open habitats ( <80% canopy cover). We infer that the differences seen between habitats were due to differences in habitat openness, and not leaf optical properties. **b** Median radiance spectra of leaf surfaces modeled with and without specular reflections when the specular light source was overlying leaves. Leaf type (deciduous or rainforest) was randomized. Here, we can see that specular reflections from the upper leaf surface shift the spectral shape of upper leaf surface reflectance more than lower leaf surface reflectance, and that this difference is more pronounced in closed habitats. The result of this in terms of median achromatic contrast across the UV-blue spectrum can be seen in the lower two panels. In closed habitats (lower right panel), specular reflections shift upper leaf surface radiance such that contrast becomes higher at longer wavelengths. Note that all habitat and environmental parameters that go unmentioned in the figure were randomized in each of 10,000 repetitions of the model

When our optical model included diffuse reflections and transmittance only, it failed to show any conditions under which the V-cone would see higher leaf-contrast than the U-cone (Fig. 7a, first panel). To increase the model’s realism, we introduced specular reflections of extended light sources. Such reflections add an even color cast to smooth objects, and should not to be confused with small glare spots like those of directly reflected sunlight (a point source). The identity of the light source that is specularly reflected (henceforth, specular light source) could be light radiating from the sky, clouds, canopy, or leaf litter.

After adding specular reflections from both upper and lower leaf surfaces, our optical model showed that the V-cone sometimes saw higher leaf-contrast than the U-cone (Fig. 7a, second panel). However, we did not see any difference in the central tendency of leaf-contrasts modeled with deciduous and rainforest leaves. When we compared leaf-contrast for leaves illuminated by different specular light sources, we found that the V-cone only saw higher leaf-contrast than the U-cone when the specular light source came from overlying leaves (Fig. 7a, third panel). Since the rainforest habitats we sampled were much denser than the deciduous ones, we inferred that the difference in optimal spectral tuning between different habitats may have stemmed from differences in the probability of overlying leaves being the specular light source. We therefore tested for an effect of canopy cover in our optical model. We found that when the canopy was >80% closed, the V-cone was more likely to see higher leaf-contrast than the U-cone (Fig. 7a, fourth panel). Conversely, when the canopy was <80% closed, the U-cone was more likely to see higher contrast than the V-cone. This suggests that canopy cover played the definitive role in the differences in optimal spectral tuning that we observed between habitats.

To better understand how specular reflections can shift optimal spectral sensitivity toward the V-cone, we plotted the median radiances of leaves modeled with and without specular reflections when the specular light source was overlying leaves (Fig. 7b). When canopy cover was >80% (Fig. 7b, top right panel), the specular light source shifted the spectral shape of upper leaf surface radiance much more than when canopy cover was <80% (Fig. 7b, top left panel). This was not true of lower leaf surfaces—specular reflections shifted mainly the intensity, and not the spectral shape, of lower leaf surface radiance, and they did so by a similar proportion under all levels of canopy cover. In short, specular reflections shifted the spectral shape of radiance differently for upper- and lower leaf surfaces, and this shift was more extreme in closed habitats. The result of this in terms of leaf-contrast from 300 to 500 nm can be seen in the bottom two panels of Fig. 7b. When canopy cover was >80%, leaf-contrast was higher above 400 nm, whereas when canopy cover was <80%, leaf-contrast was higher below 400 nm.

**Fig. 8:**
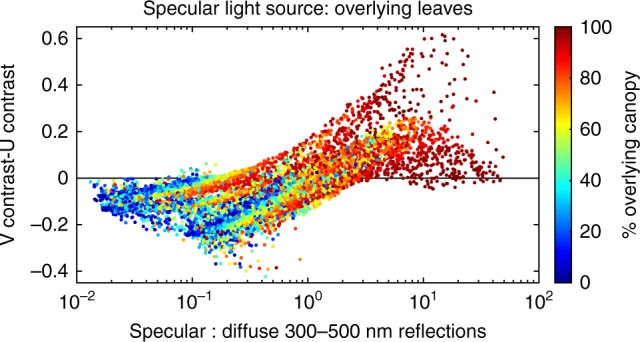
The ratio of specular to diffuse reflections from leaves affects optimal spectral tuning for perceiving leaf-contrast. The optical model revealed that optimal spectral sensitivity for visualizing leaf-contrast depends not only on the specular light source (see Fig. 7a), but also on the ratio of specular to diffuse reflections radiating from the upper leaf surface. This ratio depends on how much of the sky is blocked by overlying canopy. The less light that makes its way through holes in the overlying canopy, the greater the proportion of specular reflections relative to diffuse reflections, and the more beneficial the V-cone for visualizing leaf-contrast. The line at *y* = 0 indicates the point at which both UV-cone variants see equal contrast

It can be inferred that the reason why specular reflections shift leaf radiance more in closed than open habitats is because in closed habitats, specular reflections have a greater intensity relative to diffuse reflections. In a closed habitat, little light makes its way through holes in the canopy to contribute to diffuse reflections. As such, the ratio of specular to diffuse reflections will be higher in a closed habitat, explaining why specular reflections shift leaf radiance more in closed than open habitats. To better understand this relationship, we used our model to visualize the interrelatedness between canopy cover, the intensity of specular to diffuse reflections, and the leaf-contrast seen by the V-cone relative to the U-cone. As expected, as canopy cover increased, the proportion of specular to diffuse reflections increased, as did the leaf-contrast seen by the V-cone relative to the U-cone (Fig. 8).

Previous studies have tested which parts of the spectrum an animal should sample for optimal color discrimination in vegetated habitats. However, methodological limitations prevented them from discovering the importance of UV. Lythgoe and Partridge (1989)^17^ brought leaves into the laboratory to measure their spectral reflectance, and showed, via modeling, that a green and blue photoreceptor is the best dichromatic combination for discriminating between different green leaves. It is unclear whether they compared upper and lower leaf surfaces, but by bringing objects into the laboratory, they destroyed the effects of lighting geometry, which we discovered to be crucial for setting up strong UV leaf-contrasts. Chiao et al.^18^ used a multispectral camera to test which dichromatic combinations of photoreceptors see the greatest color contrasts in vegetated habitats, but did not sample the UV spectrum. Our study is the first to use a multi- or hyper-spectral imaging system to visualize vegetation, in situ, from an animal’s perspective, across the entire UV-visible spectrum. Because our methodology preserved natural lighting geometry and sampled the whole UV-visible spectrum, we were able to discover a phenomenon that, in retrospect, seems obvious, but which no previous study had the methodological capability or foresight to look for.

By developing an optical model to understand the physics behind our multispectral data, we discovered that specular reflections have an underappreciated role in shifting the spectral shape of light reflected by smooth objects. Studies of adaptive coloration generally do not account for specular reflections^19^. This may have led to misleading results in some cases, especially when looking at parts of the spectrum where specular reflections often dwarf diffuse ones. Our study thus opens up the possibility for a whole new realm of inquiry into the importance of specular reflections for the evolution of vision and color patterns in natural habitats.

## Methods

### Multispectral camera filter design

To calculate our target filter spectra, we calculated typical spectral sensitivities of avian cones and then divided these spectra by the spectral sensitivities/transmittances of the other components of the camera system, which was based on PIXELTEQ’s UV-VIS Spectrocam (Largo, FL, USA). Using the data tabulated in Hart and Vorobyev^6^, we calculated the mean wavelength of peak sensitivity for the opsin in each avian cone class (U, V, S, M, L), and used these wavelengths to generate opsin spectral sensitivity curves following Govardovskii et al.^20^. Next, we calculated the mean wavelength of 50% peak absorptance (*λ*_mid_) of each oil droplet class, including a separate mean for each of the two variants that can be paired with the S (blue)-cone class. For each mean *λ*_mid_, we calculated the corresponding *λ*_cut_ and then the spectral transmittance of each oil droplet using the template equations developed by Hart and Vorobyev^6^. Mean optical media transmittance spectra for birds having each variant of the UV-cone class (U and V) were calculated from the data tabulated in Lind et al.^21^.

We calculated the desired spectral sensitivity of each multispectral camera channel by multiplying each opsin curve by its associated oil droplet and ocular media transmittance spectra. For the M- and L-cones, we used the mean of the U and V optical media transmittance spectra. To obtain our target filter transmittance spectra, we divided these target spectral sensitivities by the quantum efficiency spectrum of the camera sensor (JAI CM-140 GE-UV, Kushima City, Japan), by the transmittance spectrum of the lens (Jenoptik CoastalOpt UV-VIS-IR 60 mm 1:4 Apo Macro, Jupiter, FL, USA), and by an infrared blocking filter mounted on the front of lens (commissioned from Knight Optical, Kent, UK)). Thus,

1 $$S_i\left( \lambda \right)T_i\left( \lambda \right)T_{{\mathrm{m}},i}\left( \lambda \right) = F_{{\mathrm{target}},i}\left( \lambda \right)S_{{\mathrm{sensor}}}\left( \lambda \right)T_{{\mathrm{lens}}}\left( \lambda \right)T_{{\mathrm{IRblock}}}\left( \lambda \right)$$

where

*S*_*i*_*(λ)* = spectral sensitivity of cone class *i*,

*T*_*i*_*(λ)* = spectral transmittance of oil droplet associated with cone class *i*,

*T*_m*,i*_*(λ)* = spectral transmittance of optical media (lens, cornea, vitreous fluid) associated with cone class *i*,

*F*_target*,i*_*(λ)* *=* target spectral transmittance of multispectral camera filter,

*S*_sensor_*(λ)* = spectral sensitivity of camera sensor,

*T*_lens_*(λ)* = spectral transmittance of camera lens, and

*T*_IRblock_*(λ)* = spectral transmittance of infrared blocking filter.

PIXELTEQ (Largo, FL, USA) fabricated filters according to our calculated *F*_target*,i*_*(λ)*. A comparison of avian spectral sensitivities and the effective spectral sensitivities of each camera channel is depicted in Fig. 1.

### Habitat sampling and photography

We captured 173 sets of six photographs (one through each of six filters) of terrestrial vegetated habitats, including deciduous habitats in southern Sweden (43 sets) and wet schlerophyll (50 sets) and rainforest (80 sets) habitats in Queensland, Australia. Details of field sites can be found in Supplementary Table 1. Photographs were taken at assorted field sites on calm days or portions of the day to minimize noise due to wind-induced motion of branches and leaves. Field excursions were planned to maximize the range of lighting conditions sampled, from overcast to clear skies, and from dawn to dusk. For each scene sampled, the position and direction of the camera was selected in a pseudo-random fashion designed to maximize the range of habitat and lighting conditions sampled, including, when possible, different heights in the canopy, assisted by canopy towers, walks, bridges, etc. Photographs of deciduous habitats were limited to the months of June, July, and August to avoid any confounding effects of leaf senescence. When the dynamic range of the camera was insufficient to capture much of the dynamic range of the scene, multiple sets of photographs were taken at different exposures and combined later, in MATLAB, to create high dynamic range images.

### Quantifying leaf-contrast from photographs

The output of the camera sensor of the UV-VIS Spectrocam scaled linearly with light intensity; thus, no non-linearity corrections were required. Dark noise was constant across exposures and was subtracted from all pixel values. Each pixel value of a photograph taken through a given filter represented the quantum catch by a single cone class at a single point in space. To adapt quantum catches to the intensity of the background^11^, each pixel value was normalized by the mean of all pixel values in the photograph. Each set of six photographs was then opened using the software Evince (Prediktera, Umeå, Sweden). Within this program, we hand-selected all upper leaf surfaces and all lower leaf surfaces, selecting the same pixels across all six photographs. Each species of plant was selected separately. We exported the indices of the selected pixels and then imported them into MATLAB, which we used to calculate the median quantum catch of each plant’s upper leaf surfaces and the median quantum catch of each plant’s lower leaf surfaces across each photograph. These medians were then used to calculate Michelson contrast^10^ and just noticeable differences (JNDs)^11^ between the upper and lower leaf surfaces of each plant. JNDs were calculated with log transformation of quantum catches^11^. For our comparison between LMSU and LMSV birds, Weber fractions were calculated from estimates of noise-to-signal ratios^22,23^ and cone fractions^24–28^ in terrestrially foraging birds. Weber fractions used were the following: U and V: 0.12, S: 0.092, M: 0.075, L: 0.069. For our comparison between tetrachromats and theoretical trichromats and dichromats (Fig. 5b), we wanted to maximize the generality of our findings across the animal kingdom, so we assumed that the same number of each photoreceptor class contributed to an integrative unit, and set all Weber fractions to 0.069.

If some leaves in an image were illuminated by direct sunlight, these leaves were selected and analyzed separately. If there were any over- or underexposed pixels in any channel, these pixels were excluded from all calculations across all channels. Selected normalized pixel values and their associated metadata can be downloaded as a JSON file at https://figshare.com/ under the DOI 10.6084/m9.figshare.7423532.

### Optical model input parameters

To determine typical diffuse spectral reflectances and transmittances of live leaves, we measured these properties from 17 deciduous leaves collected from the Lund University campus, and from 16 rainforest leaves collected from the Lund University Botanical Garden greenhouse in mid-June. The deciduous species sampled were: *Acer platanoides, Alnus incana, Betula pubescens, Cornus sanguinea, Corylus avellana, Crataegus laevigata, Crataegus monogyna, Fagus sylvatica, Fallopia dumetorum, Malus spp., Prunus padus, Quercus rubra, Rhamnus cathartica, Rosa spp., Sorbus aucuparia*, and *Taraxacum pallidipes*. The rainforest species sampled were: *Aglaonema nitidum, Anchomanes difformis, Arpophyllum giganteum, Artocarpus altilis, Bambusa vulgaris, Camillia japonica, Corynocarpus laevigatus, Encephalartus ferox, Epipremnum pinnatum, Myriocarpa spp., Passiflora quadrangularis, Psidium cattleianum, Syngonium podophyllum*, and *Theobromum cacao*.

To determine a typical leaf litter reflectance spectrum, we measured the diffuse spectral reflectances of both surfaces of 17 dry leaves found in leaf litter on campus and in parks in Lund, Sweden. Dead leaves were not identified to species, but visual inspection indicated that they all had come from different species.

Diffuse reflectance was measured with a reflection probe, oriented at 45° to the leaf surface, connected to a USB2000 + UV-VIS-ES spectrometer and a DH-2000 light source (both from Ocean Optics, Dunedin, FL, USA). Transmittance was measured with an integrating sphere (Electro Optical Industries Inc., Santa Barbara, CA, USA) connected to a USB2000 + UV-VIS-ES spectrometer, with a clear blue sky as a light source. For each class of spectra, we calculated the median spectrum to use as model input (Supplementary Figure 1a).

We used the American Society for Testing and Materials’ (ASTM) standard terrestrial irradiance spectra as light sources in our optical model. These include direct normal solar irradiance *I*_s_ for a sun 42° from the zenith, and hemispherical irradiance *I*_a_ for light incident on a surface tilted 37° toward the same sun under clear skies. Clear sky hemispherical irradiance *I*_b_ was calculated by subtracting *I*_s_ from *I*_a_. Overcast sky irradiance was calculated from *I*_b_ and *I*_s_ following the parameterizations of Siegel et al.^29^ using a total cloud index $\overline {CL}$ of 0.8, which is typical of overcast conditions, to calculate a spectral cloud index $\widehat {cl}$,

2 $$\widehat {cl}\left( {\lambda ;\overline {CL} } \right) = A\left( {\overline {CL} } \right)\lambda + B(\overline {CL} )$$

where $A\left( {\overline {CL} } \right)$ and $B\left( {\overline {CL} } \right)$ are defined as:

3 $$A\left( {\overline {CL} } \right) = 0.00150\overline {CL} (1 - \overline {CL} )$$

and

4 $$B\left( {\overline {CL} } \right) = 0.966\overline {CL} ^2 + 0.0619\overline {CL} - 0.0389$$

To obtain hemispherical irradiance for an overcast sky *I*_c_, the spectral cloud index $\widehat {cl}$ was multiplied by hemispherical irradiance under a clear sky^29^. Hemispherical irradiance under a clear sky *I*_cs_ was first calculated by adding clear sky irradiance *I*_b_ to solar irradiance *I*_s_ weighted by the cosine of solar zenith angle *θ*_z_,

5 $$I_{{\mathrm{cs}}}\left( \lambda \right) = I_{\mathrm{b}}\left( \lambda \right) + I_{\mathrm{s}}\left( \lambda \right)\cos {\theta _{\mathrm{z}}}$$

with *I*_c_ then calculated as:

6 $$I_{\mathrm{c}}\left( \lambda \right) = I_{{\mathrm{cs}}}\left( \lambda \right)\widehat {cl}\left( {\lambda ;\overline {CL} } \right)$$

Sun, cloud, and sky hemispherical irradiance spectra under a solar zenith angle of zero are shown in Supplementary Figure 1b. Sky and cloud radiances per steradian were estimated^10^ by dividing their irradiances by pi. Although the intensity of cloud and sky radiance can vary depending on the position of the sun and on cloud density and thickness, we found that independently varying sky and cloud radiant intensities had no major effects on the output of our optical model (i.e., the difference in leaf-contrast seen by the two avian UV-cone variants).

### Optical model calculations

The optical model was programmed to repeat its calculations of U- and V-cone leaf-contrast 10,000 times, with 12 parameters that, unless otherwise stated, were randomized each time to account for natural variation in habitat geometry and environmental conditions. Randomized parameters included (1) solar zenith angle, (2) vertical tilt of the leaves whose contrast was being calculated, (3) azimuth of the sun relative to the direction of the viewer’s gaze, (4) cloud cover, (5) whether the leaves whose contrast was being calculated were in the sun or shade, (6) proportion of the sky occluded by overlying canopy, (7) occlusion of the sun by clouds, (8) proportion of the ground (i.e., leaf litter) visible through underlying vegetation, (9) the identity of the specular light source reflected from the leaf’s upper surface (could be light radiating from the sky, clouds, or overlying canopy), (10) the identity of the specular light source reflected from the leaf’s lower surface (could be light radiating from the underlying canopy or leaf litter), (11) whether the specular light source in (10) was illuminated by direct sunlight, and (12) whether deciduous or rainforest leaf spectra were used. When each of these parameters is defined in the equations that follow, its corresponding number in this list is displayed parenthetically.

For simplicity, the avian viewer’s gaze was assumed to be horizontal, i.e., perpendicular to the direction of the zenith (Supplementary Figure 1c). The model calculated achromatic contrast between the upper and lower surfaces of two identical leaves, side by side, one with its upper surface tilted toward the observer, and the other with its upper surface tilted away from the observer. The tilt angle *θ*_l_ of the two leaves relative to the zenith was the same within a given iteration and could range between 70 and 90°. For simplicity, all other reflective surfaces in the forest (other leaves in the canopy and leaf litter) were assumed to be oriented horizontally.

When no overhead objects (canopy, clouds) blocked direct sunlight, the model calculated the angles of incidence, *θ*_s_, of the sun upon the upper surface of each of the two leaves. To do this, we followed:

7 $${\bar{\mathbf n}} = \left[ {\begin{array}{*{20}{c}} 0 & 0 & 1 \end{array}} \right]\left[ {\begin{array}{*{20}{c}} 1 & 0 & 0 \\ 0 & {\cos \theta _{\mathrm{l}}} & { - \sin \theta _{\mathrm{l}}} \\ 0 & {\sin \theta _{\mathrm{l}}} & {\cos \theta _{\mathrm{l}}} \end{array}} \right]\left[ {\begin{array}{*{20}{c}} 1 & 0 & 0 \\ 0 & {\cos 90} & { - \sin 90} \\ 0 & {\sin 90} & {\cos 90} \end{array}} \right]$$

8 $${\bar{\mathbf r}} = \left[ {\begin{array}{*{20}{c}} 0 & 0 & { - 1} \end{array}} \right]\left[ {\begin{array}{*{20}{c}} 1 & 0 & 0 \\ 0 & {\cos - \theta _{\mathrm{z}}} & { - \sin - \theta _{\mathrm{z}}} \\ 0 & {\sin - \theta _{\mathrm{z}}} & {\cos - \theta _{\mathrm{z}}} \end{array}} \right]\left[ {\begin{array}{*{20}{c}} {\cos - \gamma _{\mathrm{s}}} & { - \sin - \gamma _{\mathrm{s}}} & 0 \\ {\sin - \gamma _{\mathrm{s}}} & {\cos - \gamma _{\mathrm{s}}} & 0 \\ 0 & 0 & 1 \end{array}} \right]$$

9 $$\theta _{\mathrm{s}} = \cos ^{ - 1}\left( {{\bar{\mathbf r}} \cdot {\bar{\mathbf n}}} \right)$$

where

*θ*_z_ = solar zenith angle (parameter #1),

*θ*_l_ = tilt angle of leaf relative to the zenith (parameter #2), and,

when the upper surface of the leaf was tilted towards the observer,

$\gamma _{\mathrm{s}}$ = azimuth of the sun relative to the direction of the viewer’s gaze (parameter #3), and,

when the upper surface of the leaf was tilted away from the observer,

$$\gamma _{\mathrm{s}}$$ = $$\gamma _{\mathrm{s}} - 180^\circ$$.

If $\theta _{\mathrm{s}}$ was ≥90°, this meant that the angle of the sun relative to the leaf was such that the sun missed the leaf. When this happened, the directness of the sun’s illumination on the leaf $d$ (defined and implemented below) was set to zero.

Downwelling spectral irradiance incident upon the upper surface of the leaf tilted toward the observer was then approximated from the spectra obtained in the previous section by calculating the relative contributions of direct skylight, cloud light, sunlight, and light filtered through leaves:

10 $$I_{{\mathrm{sky}}}\left( \lambda \right) = \left( {\left( {1 - p_{\mathrm{c}}} \right) - p_{\mathrm{l}}\left( {1 - p_{\mathrm{c}}} \right)} \right)I_{\mathrm{b}}\left( \lambda \right)$$

11 $$I_{{\mathrm{cld}}}\left( \lambda \right) = \left( {p_{\mathrm{c}} - p_{\mathrm{c}}p_{\mathrm{l}}} \right)I_{\mathrm{c}}\left( \lambda \right)$$

12 $$I_{{\mathrm{sun}}}\left( \lambda \right) = dp_{\mathrm{s}}I_{\mathrm{s}}\left( \lambda \right)\cos {\theta _{\mathrm{s}}}$$

13 $$I_{\mathrm{t}}\left( \lambda \right) = p_{\mathrm{l}}\left( {\left( {1 - p_{\mathrm{c}}} \right)I_{\mathrm{b}}\left( \lambda \right) + p_{\mathrm{c}}I_{\mathrm{c}}\left( \lambda \right) + p_{\mathrm{s}}I_{\mathrm{s}}\left( \lambda \right)\cos \theta _{\mathrm{z}}} \right)T\left( \lambda \right)$$

and then summing these contributions to obtain total downwelling spectral irradiance incident upon the leaf:

14 $$I_{{\mathrm{down}}}\left( \lambda \right) = I_{{\mathrm{sky}}}\left( \lambda \right) + I_{{\mathrm{cld}}}\left( \lambda \right) + I_{{\mathrm{sun}}}\left( \lambda \right) + I_{\mathrm{t}}\left( \lambda \right)$$

where

*p*_c_ = proportion of the sky occluded by clouds (parameter #4),

*I*_b_ = downwelling irradiance of blue sky,

*I*_c_ = downwelling irradiance of cloudy sky,

*d* = indicates whether direct sunlight is blocked by leaves overhead (0 = yes, 1 = no) (parameter #5),

*I*_s_ = solar irradiance at 90° elevation,

*T* = leaf transmittance,

*p*_l_ = proportion of the sky occluded by leaves overhead (parameter #6), and

*p*_s_ = occlusion of the sun by clouds (0 = full occlusion, 1 = no occlusion) (parameter #7).

The radiance per steradian due to diffuse reflectance coming off of the leaf’s upper surface was calculated as:

15 $$L_{{\mathrm{u}},{\mathrm{d}}}\left( \lambda \right) = I_{{\mathrm{down}}}\left( \lambda \right)R_{\mathrm{u}}\left( \lambda \right){\mathrm{\pi }}^{ - 1}$$

where

*R*_u_ = diffuse reflectance of the upper leaf surface.

The radiance per steradian due to diffuse transmittance coming off of the leaf’s lower surface was calculated as:

16 $$L_{\mathrm{t}}\left( \lambda \right) = I_{{\mathrm{down}}}\left( \lambda \right)T\left( \lambda \right){\mathrm{\pi }}^{ - 1}$$

Note that the *I*_sun_ component of *I*_down_ must be calculated separately for upper leaf surface diffuse reflectance and lower leaf surface transmittance, as the angle of the sun incident upon the leaf will differ in the two cases.

The radiance per steradian due to diffuse reflectance coming off of the leaf’s lower surface was determined by first calculating upwelling irradiance as the product of downwelling irradiance and the reflectance of objects (leaves and leaf litter) below the leaf:

17 $$I_{{\mathrm{up}}}\left( \lambda \right) = \left( {I_{{\mathrm{sky}}}\left( \lambda \right) + I_{{\mathrm{cld}}}\left( \lambda \right) + \left( {1 - p_{\mathrm{l}}} \right)p_{\mathrm{s}}I_{\mathrm{s}}\left( \lambda \right)\cos {\theta _{\mathrm{z}}} + I_{\mathrm{t}}\left( \lambda \right)} \right)\\ \left( {p_{\mathrm{g}}R_{\mathrm{g}}\left( \lambda \right) + (1 - p_{\mathrm{g}})R_{\mathrm{u}}\left( \lambda \right)} \right)$$

where

*p*_g_ = proportion of the ground visible through underlying vegetation (parameter #8), and

*R*_g_ = reflectance of the leaf litter.

This upwelling irradiance was then used to calculate diffuse reflectance coming off of the lower leaf surface:

18 $$L_{{\mathrm{l}},{\mathrm{d}}}\left( \lambda \right) = I_{{\mathrm{up}}}\left( \lambda \right)R_{\mathrm{l}}\left( \lambda \right){\mathrm{\pi }}^{ - 1}$$

where

*R*_l_ = reflectance of the lower leaf surface.

To calculate spectral radiance coming off of leaves due to specular reflections, we followed the Fresnel equations^30^. We used a conservative correction factor *K* of 1 for both upper and lower surfaces; Brakke (1994)^31^ found that leaf *K* can range from 0.6 to 3.5, and that sometimes the upper surface has a greater *K* than the lower surface, and sometimes vice versa. The conclusions drawn from our model were the same regardless of where we set *K* for the upper and lower leaf surfaces within the 0.6–3.5 range. The horizontally *R*_s_ and vertically *R*_p_ polarized components of reflectance were calculated as:

19 $$R_{\mathrm{s}} = K\left( {\frac{{n_1\cos \theta _{\mathrm{l}} - n_2\sqrt {1 - \left( {\frac{{n_1}}{{n_2}}\sin \theta _{\mathrm{l}}} \right)^2} }}{{n_1\cos \theta _{\mathrm{l}} + n_2\sqrt {1 - \left( {\frac{{n_1}}{{n_2}}\sin \theta _{\mathrm{l}}} \right)^2} }}} \right)^2$$

20 $$R_{\mathrm{p}} = K\left( {\frac{{n_1\sqrt {1 - \left( {\frac{{n_1}}{{n_2}}\sin \theta _{\mathrm{l}}} \right)^2} - n_2\cos \theta _{\mathrm{l}}}}{{n_1\sqrt {1 - \left( {\frac{{n_1}}{{n_2}}\sin \theta _{\mathrm{l}}} \right)^2} + n_2\cos \theta _{\mathrm{l}}}}} \right)^2$$

The refractive index of air^32^, *n*_1_, was set to 1. The refractive index of leaves^33^, *n*_2_, was set to 1.45. Leaf refractive index was approximated as constant with wavelength as there are no published measurements or models below 400 nm. *n*_2_ may increase sharply close to 300 nm due to absorption by the leaf cuticle^34^; however, we found that setting refractive index to increase linearly from 1.45 to the highest recorded value in any natural material (4.14 at 1800 nm in Germanium)^35^ from 350 to 300 nm, or from 400 to 300 nm, had no major effects on our model’s output. In each model iteration, the specular light sources above and below (parameters #9 and #10) were extended light sources that were chosen randomly with a probability defined by the relative proportions of different objects in the habitat. If, for example, the specular light source from above was randomly selected to be overlying leaves, then the radiance of the specular light source per steradian would be calculated as:

21 $$L_{{\mathrm{sls}},{\mathrm{above}}}\left( \lambda \right) = I_{\mathrm{t}}\left( \lambda \right)(p_{\mathrm{l}}{\mathrm{\pi }})^{ - 1}$$

and the radiance per steradian of light specularly reflecting off the leaf’s upper surface would be calculated as:

22 $$L_{{\mathrm{u}},{\mathrm{s}}}\left( \lambda \right) = 0.5\left( {R_{\mathrm{s}} + R_{\mathrm{p}}} \right)L_{{\mathrm{sls}},{\mathrm{above}}}\left( \lambda \right)$$

The specular light source from below (i.e., light diffusely reflected from leaves or leaf litter) was randomly selected in a similar fashion. The radiance of this light source was calculated with the directness of sunlight (*d*_r_) on the upwelling reflective surface as a randomized parameter (0 or 1) (parameter #11), according to the proportion of the sky occluded by clouds or leaves. If, for example, leaf litter was selected as the specular light source, then the radiance of this specular light source per steradian would be calculated as:

23 $$L_{{\mathrm{sls}},{\mathrm{below}}}\left( \lambda \right) = \left( {I_{{\mathrm{sky}}}\left( \lambda \right) + I_{{\mathrm{cld}}}\left( \lambda \right) + I_{\mathrm{t}}\left( \lambda \right) + d_{\mathrm{r}}p_{\mathrm{s}}I_{\mathrm{s}}\left( \lambda \right)\cos \theta _{\mathrm{z}}} \right)R_{\mathrm{g}}\left( \lambda \right){\mathrm{\pi }}^{ - 1}$$

and the spectral radiance per steradian of light specularly reflecting from the leaf’s lower surface would be calculated as:

24 $$L_{{\mathrm{l}},{\mathrm{s}}}\left( \lambda \right) = 0.5\left( {R_{\mathrm{s}} + R_{\mathrm{p}}} \right)L_{{\mathrm{sls}},{\mathrm{below}}}\left( \lambda \right)$$

The quantum catches of cone class *i* from the upper and lower leaf surfaces, respectively, were calculated as:

25 $$Q_{i,{\mathrm{u}}} = {\int } S_i\left( \lambda \right)T_{{\mathrm{m}},i}\left( \lambda \right)\left( {L_{{\mathrm{u}},{\mathrm{d}}}\left( \lambda \right) + L_{{\mathrm{u}},{\mathrm{s}}}\left( \lambda \right)} \right)d\lambda$$

26 $$Q_{i,{\mathrm{l}}} = {\int} {S_i\left( \lambda \right)T_{{\mathrm{m}},i}\left( \lambda \right)\left( {L_{{\mathrm{l}},{\mathrm{d}}}\left( \lambda \right) + L_{\mathrm{t}}\left( \lambda \right) + L_{{\mathrm{l}},{\mathrm{s}}}\left( \lambda \right)} \right)d\lambda }$$

where

*S*_*i*_ = spectral sensitivity of cone class *i*,

*T*_m*,i*_ = transmittance spectrum of optical media (cornea, lens, vitreous) associated with cone class *i*.

These last two parameters were the same as those used to design the custom filters for the Spectrocam (see “Camera Filter Design” above). Note that U- and V-cones have transparent oil droplets, so the oil droplet transmittance spectrum has been omitted here. Michelson^10^ achromatic contrast between upper and lower leaf surfaces was calculated as:

27 $$C_i = \frac{{Q_{i,{\mathrm{u}}} - Q_{i,{\mathrm{l}}}}}{{Q_{i,{\mathrm{u}}} + Q_{i,{\mathrm{l}}}}}$$

Finally, the relative performance of the V-cone was calculated as the difference in contrast seen by the V- (*C*_V_) and U- (*C*_U_) cones:

28 $$R = C_{\mathrm{V}} - C_{\mathrm{U}}$$

*R* *>* 0 indicates that the V-cone would see higher leaf-contrast, *R* < 0 indicates that the U-cone would see higher leaf-contrast, and *R* = 0 indicates that the V- and U-cones would see equal leaf-contrast.

### Code availability

The MATLAB code used to execute the analyses is available from the corresponding author upon request.

### Reporting summary

Further information on experimental design is available in the Nature Research Reporting Summary linked to this article.

## Supplementary information

Supplementary Information

Reporting Summary

## Data Availability

The source data underlying Figs. 5–6 and Tables 1–2 can be found at https://figshare.com/ under the DOI 10.6084/m9.figshare.7423532. A reporting summary for this Article is available as a Supplementary Information file.

**Table 2:** Details of sign tests used to analyze data in Fig. 6

Null hypotheses tested	Deciduous (*N* = 95) sign statistic, *P*-value	Wet schlerophyll (*N* = 109) sign statistic, *P*-value	Rainforest (*N* = 161) sign statistic, *P*-value
V-contrast−U-contrast = 0	38, 0.06	70, 0.004	122, <0.0001
S(V)-contrast−S(U)-contrast = 0	3, <0.0001	1, <0.0001	12, <0.0001
LMSV−LMSU = 0	19, <0.0001	31, <0.0001	72, 0.2

The sign statistic indicates the number of times the difference in leaf-contrast seen by different channels or combinations of channels (shown in the null hypotheses tested column) was greater than zero Details of sign tests used to analyze data in Fig. 6 The sign statistic indicates the number of times the difference in leaf-contrast seen by different channels or combinations of channels (shown in the null hypotheses tested column) was greater than zero
