# Supplementary material for: Avian UV vision enhances leaf surface contrasts in forest environments
Source: Nat Commun. 2019 Jan 22;10:238. doi: 10.1038/s41467-018-08142-5 (PMC6342963; doi:10.1038/s41467-018-08142-5)
Supplement: Supplementary file 1 — Supplementary Information [file 41467_2018_8142_MOESM1_ESM.pdf]

Supplementary Information

Avian UV vision enhances leaf surface contrasts in forest environments

Tedore & Nilsson

Supplementary Table 1. Field sites.

habitat	climate zone	nature reserve or nearest landmark	GPS (approximate)	nearest city/locality, country	N image sets	N leaf contrasts
deciduous	temperate	Brunnshögs kojskog	55.718550, 13.237920	Lund, Skåne, Sweden	2	2
deciduous	temperate	Dalby Norreskog	55.682585, 13.338666	Dalby, Skåne, Sweden	2	2
deciduous	temperate	Dalby Söderskog	55.676308, 13.330033	Dalby, Skåne, Sweden	4	4
deciduous	temperate	Ekologihuset, Lunds Universitet	55.713641, 13.208902	Lund, Skåne, Sweden	15	51
deciduous	temperate	Nöbbelövs mosse	55.734447, 13.154098	Lund, Skåne, Sweden	1	2
deciduous	temperate	Skrylle naturreservat	55.692614, 13.360024	Södra Sandby, Skåne, Sweden	17	31
wet schlerophyll	subtropical	Lower Bellbird, Lamington National Park	-28.194903, 153.188794	Binna Burra, Queensland, Australia	2	3
wet schlerophyll	subtropical	Caves Circuit, Lamington National Park	-28.194903, 153.188794	Binna Burra, Queensland, Australia	1	2
wet schlerophyll	tropical	Atherton Tablelands Birdwatchers' Cabin (114 Webster Rd)	-17.442594, 145.456770	Wondecla, Queensland, Australia	47	117
rainforest	subtropical	Border Track, Lamington National Park	-28.194903, 153.188794	Binna Burra, Queensland, Australia	3	3
rainforest	subtropical	Bellbird Clearing, Lamington National Park	-28.194903, 153.188794	Binna Burra, Queensland, Australia	1	2
rainforest	subtropical	Mick's Tower, Lamington National park	-28.230772, 153.135947	O'Reilly, Queensland, Australia	7	20
rainforest	tropical	Mamu Tropical Skywalk	-17.612999, 145.798213	Mamu, Queensland, Australia	12	20
rainforest	tropical	Kuranda Birdwatchers' Cabin (25 Butler Drive)	-16.805068, 145.637177	Kuranda, Queensland, Australia	19	37
rainforest	tropical	Jindalba Boardwalk, Daintree National Park	-16.238442, 145.432449	Cow Bay, Queensland, Australia	3	4
rainforest	tropical	Daintree Discovery Center	-16.237519, 145.427818	Cow Bay, Queensland, Australia	8	15
rainforest	tropical	Thornton Beach Bungalows (Lot 1 Cape Tribulation Rd)	-16.171935, 145.441511	Thornton Beach, Queensland, Australia	1	1
rainforest	tropical	Marrdja Boardwalk, Daintree National Park	-16.137991, 145.440460	Cape Tribulation, Queensland, Australia	1	2
rainforest	tropical	Mount Hypipamee National Park	-17.428063, 145.486096	Wondecla, Queensland, Australia	25	57

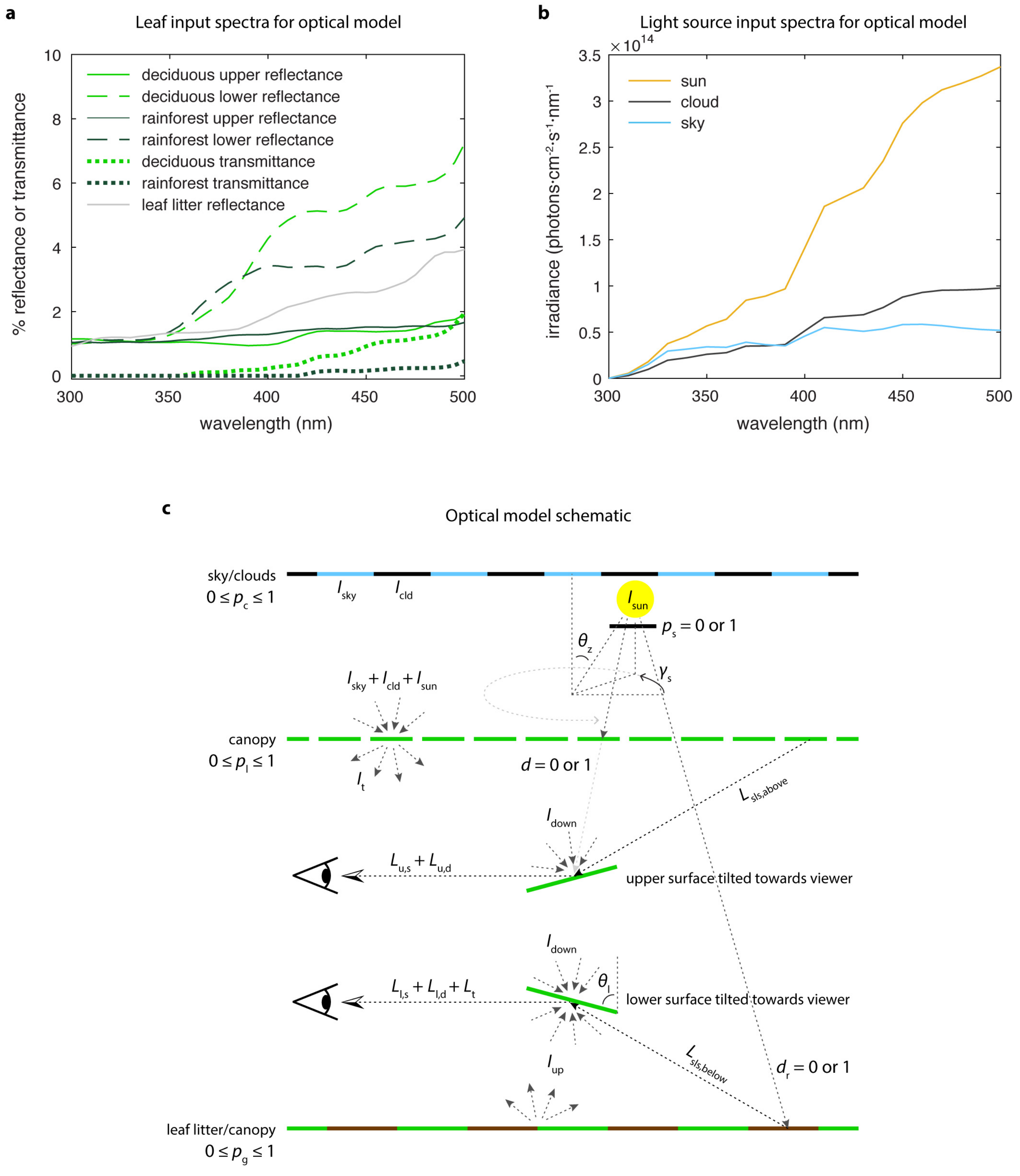

Supplementary Figure 1. Optical model input spectra and schematic. (a) Median diffuse reflectances and transmittances of leaves and leaf litter. (b) Irradiances of light sources at a solar zenith angle of zero. (c) Optical model schematic showing habitat geometry and several model parameters.
