# Supplementary material for: Avian UV vision enhances leaf surface contrasts in forest environments
Source: Nat Commun. 2019 Jan 22;10:238. doi: 10.1038/s41467-018-08142-5 (PMC6342963; doi:10.1038/s41467-018-08142-5)
Supplement: Supplementary file 2 — Reporting Summary [file 41467_2018_8142_MOESM2_ESM.pdf]

nature research

Corresponding author(s): Cynthia Tedore

Reporting Summary

Nature Research wishes to improve the reproducibility of the work that we publish. This form provides structure for consistency and transparency in reporting. For further information on Nature Research policies, see Authors & Referees and the Editorial Policy Checklist.

Statistical parameters

When statistical analyses are reported, confirm that the following items are present in the relevant location (e.g. figure legend, table legend, main text, or Methods section).

n/a	Confirmed	
☐	☒	The exact sample size (n) for each experimental group/condition, given as a discrete number and unit of measurement
☐	☒	An indication of whether measurements were taken from distinct samples or whether the same sample was measured repeatedly
☐	☒	The statistical test(s) used AND whether they are one- or two-sided<br>*Only common tests should be described solely by name; describe more complex techniques in the Methods section.*
☐	☒	A description of all covariates tested
☐	☒	A description of any assumptions or corrections, such as tests of normality and adjustment for multiple comparisons
☐	☒	A full description of the statistics including central tendency (e.g. means) or other basic estimates (e.g. regression coefficient) AND variation (e.g. standard deviation) or associated estimates of uncertainty (e.g. confidence intervals)
☐	☒	For null hypothesis testing, the test statistic (e.g. F, t, r) with confidence intervals, effect sizes, degrees of freedom and P value noted<br>*Give P values as exact values whenever suitable.*
☒	☐	For Bayesian analysis, information on the choice of priors and Markov chain Monte Carlo settings
☒	☐	For hierarchical and complex designs, identification of the appropriate level for tests and full reporting of outcomes
☒	☐	Estimates of effect sizes (e.g. Cohen's d, Pearson's r), indicating how they were calculated
☐	☒	Clearly defined error bars<br>*State explicitly what error bars represent (e.g. SD, SE, CI)*

Our web collection on statistics for biologists may be useful.

Software and code

Policy information about availability of computer code

Data collection	Spectrocam image acquisition was managed using a laptop running the Pixelteq Spectrocam Software.
Data analysis	All analyses were performed using MATLAB 2017b.

For manuscripts utilizing custom algorithms or software that are central to the research but not yet described in published literature, software must be made available to editors/reviewers upon request. We strongly encourage code deposition in a community repository (e.g. GitHub). See the Nature Research guidelines for submitting code & software for further information.

Data

Policy information about availability of data

All manuscripts must include a data availability statement. This statement should provide the following information, where applicable:

- Accession codes, unique identifiers, or web links for publicly available datasets
- A list of figures that have associated raw data
- A description of any restrictions on data availability

The source data underlying Figs. 5-6 and Tables 1-2 can be found at https://figshare.com/ under the DOI 10.6084/m9.figshare.7423532.

Field-specific reporting

Please select the best fit for your research. If you are not sure, read the appropriate sections before making your selection.

☐ Life sciences ☐ Behavioural & social sciences ☒ Ecological, evolutionary & environmental sciences

For a reference copy of the document with all sections, see nature.com/authors/policies/ReportingSummary-flat.pdf

Ecological, evolutionary & environmental sciences study design

All studies must disclose on these points even when the disclosure is negative.

Study description	We took multispectral photographs of three types of vegetated habitats: 1) deciduous, 2) wet schlerophyll, and 3) rainforest.
Research sample	Photographs in each habitat were taken across varied field sites. Sampling was particularly abundant at large sites that were easy to access from wherever CT was based during field work.
Sampling strategy	During field excursions, we recorded cloud cover, occlusion of the sun by clouds, and sun elevation. We stopped collecting data once we had obtained a relatively even distribution of data across these environmental conditions in each habitat.
Data collection	CT collected the data using Pixelteq's UV-VIS Spectrocam connected to a laptop running Pixelteq's Spectrocam image acquisition software.
Timing and spatial scale	Photographs of deciduous habitats were taken on June 21, 27, 28 and July 1, 2, 4, 17 in 2015, and on July 5, 20 and August 16, 19, 21 in 2016. Photographs of wet schlerophyll habitats were taken on August 17, 20 and September 8, 9 in 2015, and March 12-15, 19, 20 in 2016. Photographs of rainforest habitats were taken on August 19-21, 23, 26, 28, 30, 31, and September 1, 6, 7, 11 in 2015, and on March 9-11, 15, 18-22 in 2016. The maximum distance between deciduous sites was 20 km, between wet schlerophyll sites was 1440 km, and between rainforest sites was 1560 km.
Data exclusions	No data were excluded from analyses.
Reproducibility	We bootstrapped our data to check the repeatability of our findings. During 100 bootstraps, 3 out of 55 significant differences sometimes became non-significant. The difference between V- and U-contrast become non-significant in wet schlerophyll habitats 14 out of 100 times, the difference between LMU and LMS contrast became non-significant in rainforest habitats 19 out of 100 times, and the difference between MU and MS contrast became non-significant in rainforest habitats 40 out of 100 times. Also during these 100 bootstraps, two out of two non-significant relationships sometimes became significant. U-contrast became significantly greater than V-contrast in deciduous habitats 47 out of 100 times, and LMSU contrast became significantly greater than LMSV contrast in rainforest habitats 14 out of 100 times.
Randomization	Photographed sites and the position and orientation of the camera in each site were selected in a pseudo-random fashion designed to maximize the range of habitat and environmental conditions sampled.
Blinding	Blinding was not relevant to our study because it was open-ended with no a priori hypotheses, and because data (in the form of pixel values) were already in a numeric format from the moment each photo was taken.

Did the study involve field work? ☒ Yes ☐ No

Field work, collection and transport

Field conditions	Photographs were taken throughout the day, from shortly before sunrise till shortly after sunset, with sun elevations ranging from -0.44-76°, and under varying levels of cloud cover, ranging from overcast to clear. Photos were not taken when vegetation was visibly wet, nor were they taken during foggy conditions.
Location	Detailed location information can be found in Supplementary Table 1.
Access and import/export	No permits were required, as field work was limited to taking photographs.
Disturbance	No disturbance was caused by the study.

Reporting for specific materials, systems and methods

Materials & experimental systems

n/a	Involved in the study
☒	☐ Unique biological materials
☒	☐ Antibodies
☒	☐ Eukaryotic cell lines
☒	☐ Palaeontology
☒	☐ Animals and other organisms
☒	☐ Human research participants

Methods

n/a	Involved in the study
☒	☐ ChIP-seq
☒	☐ Flow cytometry
☒	☐ MRI-based neuroimaging
